# Supplementary material for: Distinct proteomic signatures in Ethiopians predict acute and long-term sequelae of COVID-19
Source: Front Immunol. 2025 May 22;16:1575135. doi: 10.3389/fimmu.2025.1575135 (PMC12137110; doi:10.3389/fimmu.2025.1575135)
Supplement: Supplementary file 1 [file DataSheet1.pdf]

Supplemental materials

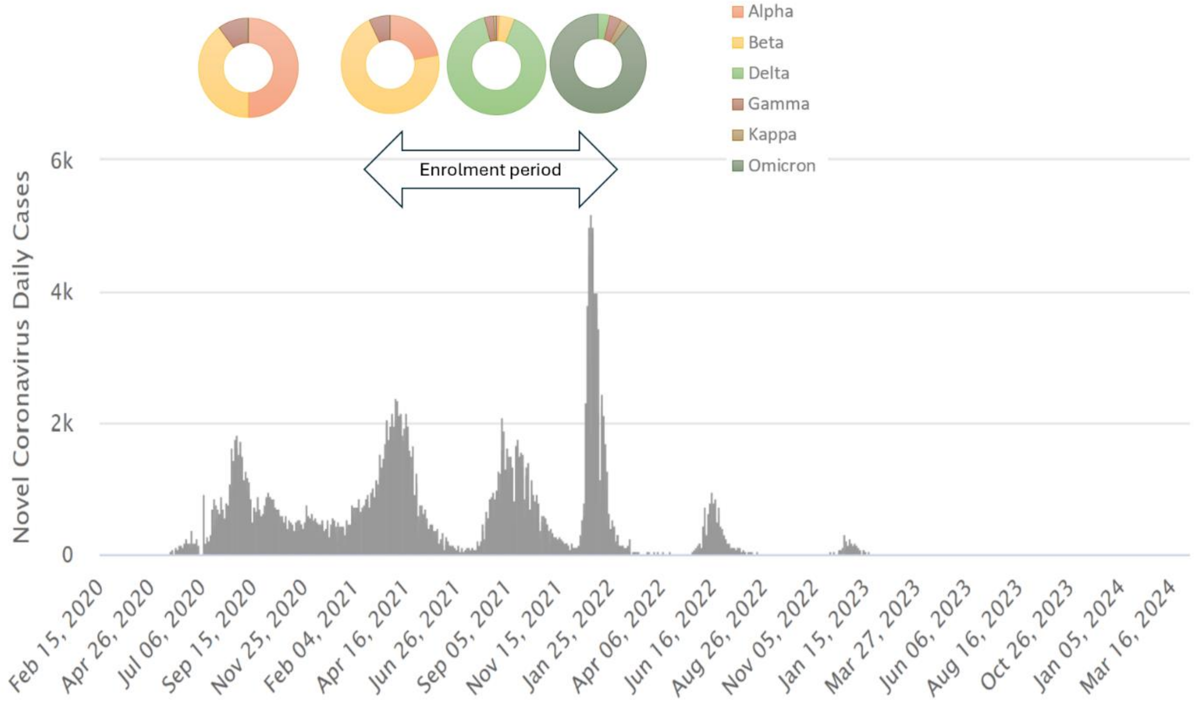

Supplementary Figure 1. The four major COVID-19 waves in Ethiopia (**Source: https://www.worldometers.info/coronavirus/country/ethiopia**) and the corresponding SARS-CoV-2 variants circulating in the country during the different pandemic waves. Data for the SARS-CoV-2 genotyping is obtained from EPHI and has been published previously (44-46). The cohort for the current study enrolled between waves 2 and 4.

Supplementary Figure 2. Gene Ontology (GO), KEGG and Reactome pathways (A) and STRING protein-protein interaction (B).

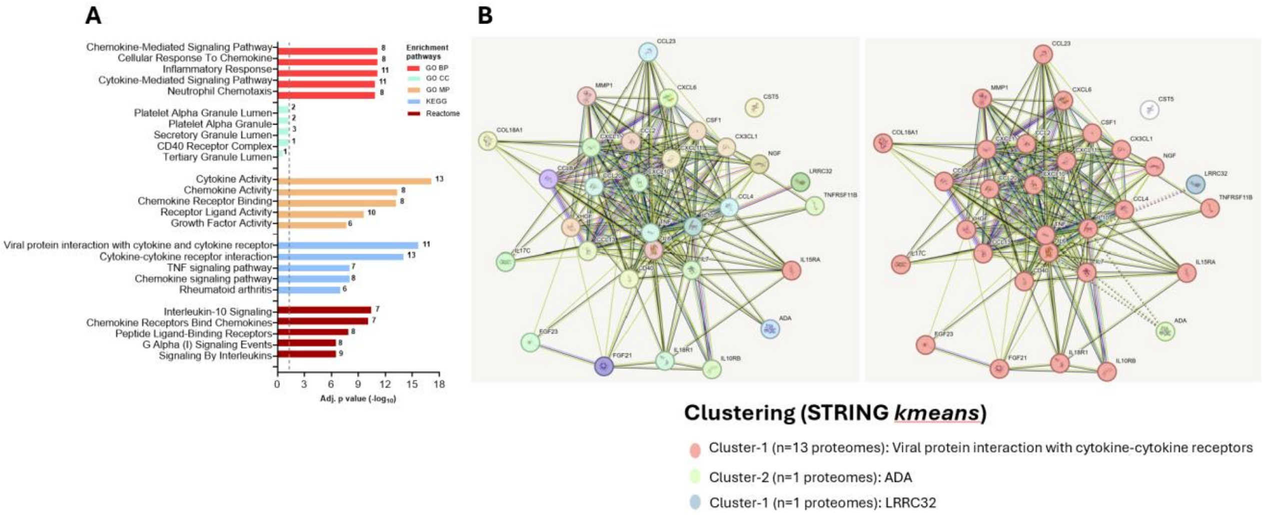

Supplementary Table 1. Baseline Clinical Characteristics of Study Participants

**Characteristic [a]**	**All COVID-19 (n =67)**	**Non-critical COVID-19 (n =45)**	**Critical COVID-19 (n =22)**	***P* value***
Headache	25 (45.5)	11 (33.3)	14 (63.6)	**0.027**
Loss of smell	27 (49.1)	12 (36.4)	15 (68.2)	**0.021**
Loss of taste	25 (45.5)	11 (33.3)	14 (63.6)	**0.027**
Cough	22 (40.0)	5 (15.2)	17 (77.3)	**<0.0001**
Shortness of breath	17 (30.9)	4 (12.1)	13 (59.1)	**<0.0001**
Wheezing	13 (23.6)	6 (18.2)	7 (31.8)	0.244
Chest pain	22 (40.0)	9 (27.3)	13 (59.1)	**0.018**
Sore throat	26 (47.3)	9 (27.3)	17 (77.3)	**<0.0001**
Nasal congestion	25 (45.5)	13 (39.4)	12 (54.6)	0.269
Anorexia	25 (45.5)	14 (42.2)	11 (50.0)	0.580
Vomiting/nausea	12 (21.8)	11 (33.3)	1 (4.6)	**0.011**
Diarrhea	8 (14.6)	7 (21.2)	1 (4.6)	0.086
Abdominal pain	9 (16.4)	7 (21.2)	2 (9.1)	0.234
Arthralgia	29 (52.7)	13 (39.4)	16 (72.7)	**0.015**
Myalgia	28 (50.9)	12 (36.4)	16 (72.7)	**0.008**
Fatigue or malaise	30 (54.6)	13 (39.4)	17 (77.3)	**0.006**
Fever	28 (50.9)	12 (36.4)	16 (72.7)	**0.008**
Conjunctivitis	18 (32.7)	15 (45.5)	3 (13.6)	**0.014**
Pneumonia	15 (27.3)	0 (0.0)	15 (68.2)	**<0.0001**

[a]Data is number (%). *Differences calculated using Pearson's Chi2 or Fisher's exact test where appropriate.

Supplementary Table 2. Inflammatory panel proteomics assessed in this study.

ADA	Adenosine Deaminase
ARTN	Artemin
AXIN1	Axin-1
Beta-NGF	Beta-nerve growth factor
CASP-8	Caspase-8
CCL9	C-C motif chemokine 19
CCL20	C-C motif chemokine 20
CCL23	C-C motif chemokine 23
CCL25	C-C motif chemokine 25
CCL28	C-C motif chemokine 28
CCL3	C-C motif chemokine 3
CCL4	C-C motif chemokine 4
CD40	CD40L receptor
CDCP1	CUB domain-containing protein 1
CXCL1	C-X-C motif chemokine 1
CXCL10	C-X-C motif chemokine 10
CXCL11	C-X-C motif chemokine 11
CXCL5	C-X-C motif chemokine 5
CXCL6	C-X-C motif chemokine 6
CXCL9	C-X-C motif chemokine 9
CST5	Cystatin D
DNER	Delta and Notch-like epidermal growth factor-related receptor
CCL11	Eotaxin
4E-BP1	Eukaryotic translation initiation factor 4E-binding protein 1
FGF-19	Fibroblast growth factor 19
FGF-21	Fibroblast growth factor 21
FGF-23	Fibroblast growth factor 23
FGF-5	Fibroblast growth factor 5
Flt3L	Fms-related tyrosine kinase 3 ligand
CX3CL1	Fractalkine
GDNF	Glial cell line-derived NEUtrophic factor
HGF	Hepatocyte growth factor
IFN-gamma	Interferon gamma
IL-1 alpha	Interleukin-1 alpha
IL10	Interleukin-10
IL-10RA	Interleukin-10 receptor subunit alpha
IL-10RB	Interleukin-10 receptor subunit beta
IL-12B	Interleukin-12 subunit beta
IL-13	Interleukin-13
IL-15RA	Interleukin-15 receptor subunit alpha
IL-17A	Interleukin-17A
IL-17C	Interleukin-17C
IL-18	Interleukin-18
IL-18R1	Interleukin-18 receptor 1
IL-2	Interleukin-2
IL-2RB	Interleukin-2 receptor subunit beta
IL-20	Interleukin-20
IL-20RA	Interleukin-20 receptor subunit alpha
IL-22 RA1	Interleukin-22 receptor subunit alpha-1
IL-24	Interleukin-24

IL-33	Interleukin-33
IL-4	Interleukin-4
IL5	Interleukin-5
IL6	Interleukin-6
IL-7	Interleukin-7
IL-8	Interleukin-8
LAP TGF-beta-1	Latency-associated peptide transforming growth factor beta-1
LIF	Leukemia inhibitory factor
LIF-R	Leukemia inhibitory factor receptor
CSF-1	Macrophage colony-stimulating factor 1
MMP-1	Matrix metalloproteinase-1
MMP-10	Matrix metalloproteinase-10
MCP-1	Monocyte chemotactic protein 1
MCP-2	Monocyte chemotactic protein 2
MCP-3	Monocyte chemotactic protein 3
MCP-4	Monocyte chemotactic protein 4
CD244	Natural killer cell receptor 2B4
NT-3	NEUtrophin-3
NRTN	Neurturin
OSM	Oncostatin-M
OPG	Osteoprotegerin
PD-L1	Programmed cell death 1 ligand 1
EN-RAGE	Protein S100-A12
SLAMF1	Signaling lymphocytic activation molecule
SIRT2	SIR2-like protein 2
STAMPB	STAM-binding protein
SCF	Stem cell factor
ST1A1	Sulfotransferase 1A1
CD6	T cell surface glycoprotein CD6 isoform
CD5	T-cell surface glycoprotein CD5
CD8A	T-cell surface glycoprotein CD8 alpha chain
TSLP	Thymic stromal lymphopoietin
TNFB	TNF-beta
TRANCE	TNF-related activation-induced cytokine
TRAIL	TNF-related apoptosis-inducing ligand
TGF-alpha	Transforming growth factor alpha
TWEAK	Tumor necrosis factor Ligand superfamily member 12
TNF	Tumor necrosis factor
TNFSF14	Tumor necrosis factor ligand superfamily member 14
TNFRSF9	Tumor necrosis factor receptor superfamily member 9
uPA	Urokinase-type plasminogen activator
VEGF-A	Vascular endothelial growth factor A
